# Evaluating Alternatives to Locomotion Scoring for Lameness Detection in Pasture-Based Dairy Cows in New Zealand: Infra-Red Thermography

**DOI:** 10.3390/ani11123473

**Published:** 2021-12-06

**Authors:** Chacha Wambura Werema, Linda Laven, Kristina Mueller, Richard Laven

**Affiliations:** 1 School of Veterinary Science, Massey University, Private Bag 11 222, Palmerston North 4442, New Zealand; L.J.Laven@massey.ac.nz (L.L.); K.Mueller@massey.ac.nz (K.M.); R.Laven@massey.ac.nz (R.L.); 2 College of Veterinary Medicine and Biomedical Sciences, Sokoine University of Agriculture, Morogoro 67 115, Tanzania

**Keywords:** lameness, infrared thermography, locomotion scoring, dairy cows, pasture-based

## Abstract

**Simple Summary:**

Early detection accompanied by effective treatment is vital to minimise the negative impacts of lameness in dairy cows. Locomotion scoring is commonly used for detecting lameness but can be challenging to implement effectively in cows at pasture-based systems. One potential alternative detection is measuring foot skin temperature using an infrared camera. Data were collected from a 940-cow dairy farm in New Zealand with cows observed at two consecutive afternoon milkings. Locomotion scoring was undertaken at the first milking and thermal imaging of the hind feet at the second milking. As the locomotion score increased, mean foot skin temperature increased, showing that measuring temperature could be a useful alternative to locomotion scoring. However, the process needs to be speeded up and automated if it is to be used widely.

**Abstract:**

Lameness in cattle is a complex condition with huge impacts on welfare, and its detection is challenging for the dairy industry. The present study aimed to evaluate the association between foot skin temperature (FST) measured using infrared thermography (IRT) and locomotion scoring (LS) in dairy cattle kept at pasture. Data were collected from a 940-cow dairy farm in New Zealand. Cows were observed at two consecutive afternoon milkings where LS was undertaken at the first milking (4-point scale (0–3), DairyNZ). The next day, cows were thermally imaged from the plantar aspect of the hind feet using a handheld T650sc forward-looking infrared camera (IRT). The association between FST and locomotion score was analysed using a generalised linear model with an identity link function and robust estimators. ROC curves were performed to determine optimal threshold temperature cut-off values by maximising sensitivity and specificity for detecting locomotion score ≥ 2. There was a linear association between individual locomotion scores and FST. For mean temperature (MT), each one-unit locomotion score increase was associated with a 0.944 °C rise in MT. Using MT at a cut-off point of 34.5 °C produced a sensitivity of 80.0% and a specificity of 92.4% for identifying cows with a locomotion score ≥ 2 (lame). Thus, IRT has a substantial potential to be used on-farm for lameness detection. However, automation of the process will likely be necessary for IRT to be used without interfering with farm operations.

## 1. Introduction

Lameness is a complex multifactorial condition characterised by an abnormal gait, pain, and discomfort. Research shows that, in addition to its major impact on dairy cow welfare [1,2,3], lameness is also responsible for substantial economic losses due to treatment costs [4], reduced milk production [5,6,7] and reproductive performance [8,9,10], and increased culling [10,11,12,13,14]. Therefore, early detection and treatment of lame cows are vital to minimise the pain and discomfort associated with lameness [15,16] and to reduce the risk of irreversible claw damage [17]. Thus, early intervention improves welfare and decreases the economic impact of lameness but requires active lameness detection. 

Locomotion scoring (LS) is the most used method for detecting lameness on dairy farms. An observer rates the cow with a discrete score based on assessing various features of gait and posture. Numerous LS systems have been developed for use on-farm; Schlageter-Tello et al. [18] identified 25 different LS systems that had been published in the peer-reviewed literature by 2014. These systems vary in the features they use. For example, while Manson and Leaver [19] included the ability of a cow to turn, Sprecher et al. [10] did not, with the opposite being true for arching of the back. LS systems also vary in the scale they use, ranging from a simple two-point system (sound or lame) [20] to as many as nine points [19,21]. The most commonly cited system is the 5-point (1 to 5) system proposed by Sprecher et al. [10] as reported by Schlageter-Tello et al. [18]. While this system has been used in New Zealand [22], the current industry standard scheme is a 4-point (0 to 3) system based on a similar system used in the UK [23]. 

The large number of different LS systems identified by Schlageter-Tello et al. [18] shows no consensus on the optimal system, with each system having its own advantages and disadvantages. One crucial problem is the subjective nature of LS, with both within- and between-observer variation being high, especially when training is limited [18,24,25,26]. Visual LS is also time-consuming and can require significant labour resources, especially in larger herds where the rate at which cows exit the milking parlour can add significant difficulties for LS. This issue led Ranjbar et al. [27], who investigated risk factors for lameness in Australian dairy herds, to record locomotion scores as a tally rather than at the individual cow level. Furthermore, in pasture-based systems where LS is usually undertaken outside as cows return to pasture after milking, environmental factors such as sunlight, wind, and rain can make LS more difficult for the observer and, therefore, less accurate. 

One potential alternative to visual LS is infrared thermography (IRT), a non-invasive technique that measures body surface temperature and produces a pictographic representation of the imaged structure [28]. The infrared camera absorbs infrared radiation and generates an image derived from the amount of heat produced. Each pixel in the produced image represents the recorded surface temperature of the anatomical region [29]. The images can be presented both in greyscale and colour. When presented in the greyscale or colour, white or red are the hottest region, whereas black or blue represent the coldest region [28,30]. 

Extremities’ and surface skin temperature mostly depends on blood perfusion and tissue metabolism rate [31]. Changes in blood flow can impact the amount of radiated heat and therefore be detected by IRT [28]. One of the key reasons for changes in tissue blood flow is the inflammatory process. This link between inflammation and tissue temperature has stimulated the use of IRT as a diagnostic tool for lameness. However, inflammation is not the only process affecting foot temperature. Other factors, such as individual animal variation, physiological state, environment, and even activity level, can influence foot temperature [32,33,34]. For example, foot temperatures measured at the coronary band are higher in early to mid-lactation (≤200 days in milk) compared to late lactation (>200 days in milk) [32,34,35]. All these factors need to be considered when evaluating the utility of IRT as a method of lameness detection in dairy cattle.

Nevertheless, there is a definite potential for IRT to be used for both screening for lameness and monitoring after treatment. For example, Wood et al. [36] recorded foot temperature fortnightly at milking using a non-contact infra-red thermometer alongside LS. They found that foot temperature was highest when a cow was identified as lame. Treatment resulted in a marked reduction in foot temperature, with the lowest foot temperature recorded six weeks after treatment. They noted that this temperature was lower than the temperature recorded six weeks before treatment, suggesting that an inflammatory process had been present in the foot for at least six weeks before detecting lameness using LS.

There is increasing interest in the use of IRT to detect lameness in dairy cows. This increase may be related to the reducing costs of IRT and continued technical advances, which has meant that IRT has become affordable [37,38]. However, almost all the published studies of lameness and IRT have been undertaken in housed cows rather than in cows kept permanently at pasture, the production system that predominates in New Zealand. Furthermore, as the environment influences foot temperature [39,40], animal activity [33], and the type of lesion that is likely to be causing lameness [41,42], the relationship between IRT and lameness may be different in pasture-based systems. 

Of the peer-reviewed studies looking at IRT and lameness detection, all the papers that include data from cattle at pasture also include data from housed cows. As far as the authors know, no peer-reviewed study has analysed pasture-based dairy cows data separately from housed dairy cows data. For example, some of the data evaluated by Rodríguez et al. [43] came from cows at pasture during spring and early summer (September to January). However, they also included data from cows housed for winter (June-August) and did not differentiate between the two groups. Similarly, Harris-Bridge et al. [44] included data from cattle that were allowed to graze during the day in spring and summer (March to August) as well as from cattle that were housed during the winter or which were permanently housed but did not include housing status as a variable in their analyses.

Further data on the association between IRT and lameness are needed in cattle at pasture. This is particularly relevant for New Zealand, as cattle are kept at pasture and never housed on the great majority of farms. So, we hypothesised that measuring the foot skin temperature from the plantar aspect of the hind feet of dairy cows would predict higher locomotion scores (lameness). Therefore, this study aimed to evaluate the association between hindfoot skin temperature, measured using IRT and LS in New Zealand dairy cattle kept permanently at pasture. 

## 2. Materials and Methods

### 2.1. Animals and Farm Location

The study was undertaken in February on a 940-cow dairy farm in the Tararua district of the North Island of New Zealand. The farmer was a client of the Massey Farm Practice and when informed about this project was interested in participating. The herd was a split calving herd with 480 cows calving in the Spring (July–October) and 460 cows in the Autumn (March–May). Most of the cows were Friesian, with approximately 20% Jersey and 5% Friesian cross Jersey. The cows were of mixed ages ranging from 2 to 10 years with four years on average. 

The cows were milked twice daily through a 60-unit rotary milking parlour. The milking herd was managed as two roughly equal groups, grazing separate paddock rotations and milking in succession. Lame cows were generally identified by farm staff and then presented for treatment by a veterinarian. Cows identified as lame by farm staff were kept in a separate “lame group” in paddocks near the milking parlour until their lameness had improved enough to return to their main herd section. The lame group was excluded from the present study as, to minimise walking and standing time, cows in the group were milked only in the morning session. Based on farm treatment records of 50 lameness cases over the lactation, the main causes of lameness were white line disease (54%), sole injury (16%) and foot rot (8%). No digital dermatitis was identified at any time.

Data collection involved observation of cows at two consecutive afternoon milkings. Locomotion scoring was undertaken at the first milking, with IRT being used at the second.

### 2.2. Locomotion Scoring

Individuals were identified by their ear tag number and locomotion scored as they exited the milking parlour by CWW using the DairyNZ lameness score [45]. This scoring system has been adapted from the Agriculture and Horticulture Development Board (AHDB) mobility score to create a system that can be used to score cattle when they are walking back to pasture after being milked [23]. The DairyNZ lameness score is based on assessing walking speed, walking rhythm, weight-bearing, back alignment, head position, stride length, and foot placement (Table 1). 

Prior to the study commencing in February 2018, CWW was trained in locomotion scoring. The training consisted of observing training videos created by DairyNZ [46] and AHDB [47], followed by supervised locomotion scoring on-farm (live cows) with a trained and experienced observer until the trainer was satisfied that the trainee could perform locomotion scoring effectively. 

Visit 1: The whole herd was locomotion scored as they exited the milking parlour after afternoon milking. Locomotion scores were recorded at the individual cow level; the score was not recorded if a cow could not be identified from its ear tag. The locomotion scoring evaluation area was a flat concrete surface about 20 m in length. This walking distance was enough to assess cows’ gait and posture attributes while exiting the milking parlour.

### 2.3. Infrared Thermography

Visit 2: Infrared thermography (IRT) imaging was performed during the next afternoon milking using a handheld T650sc Forward-looking Infrared camera (FLIR Systems, Wilsonville, OR, USA). On this day, the recorded atmospheric temperature was 22 °C. The infrared camera employed in this study had the emissivity value set at 0.95 (this relates to the capability of the object or body to absorb and emit infrared radiation).

During this visit, the speed of the rotary platform was reduced to allow routine herd pregnancy diagnosis to be undertaken. CWW performed infrared thermography imaging of the claws in the hind feet at the three-quarter point on the rotation towards the exit before cows were pregnancy tested by another veterinarian. With the observer stationary (at a distance of approximately 1 metre from the cow) and the platform rotating, a plantar image of both hind feet was obtained of every fourth cow and her identity recorded. No claw preparation was performed, feet were not washed before imaging. 

**Figure 1:**
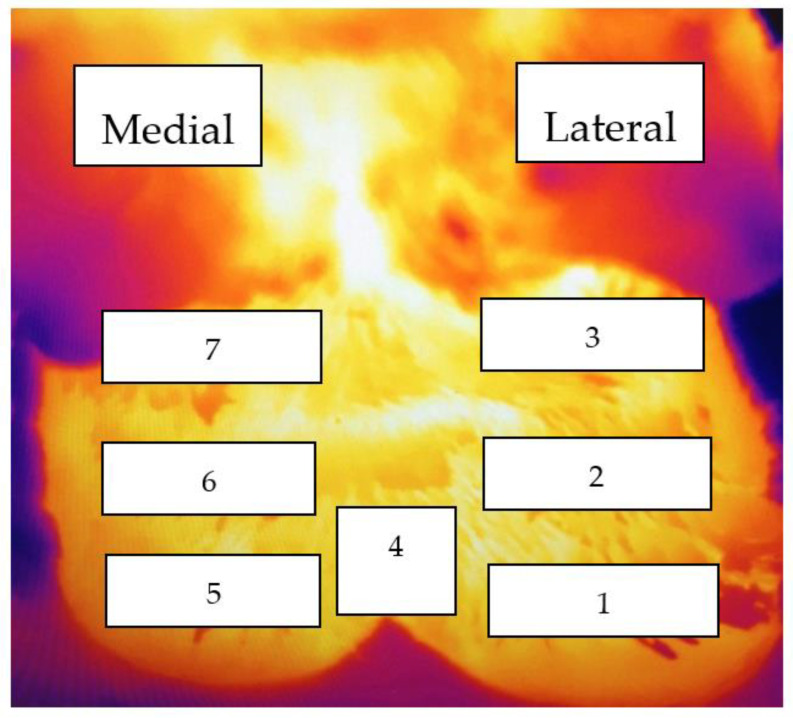
Infrared thermography image of the plantar aspect of the right hind foot overlaid to illustrate the seven zones for which estimates of surface temperature were obtained. On the lateral claw; zone 1: coronary band (CB), zone 2: above the coronary band (ACB), zone 3: below accessory digit (BAD), zone 4: interdigital space (IDS), zones 5 to 7 are the equivalent to zones 1 to 3 but on the medial claw.

The foot images were later analysed using FLIR Tools software (FLIR Systems, Wilsonville, OR, USA). The surface temperature estimates were obtained from seven zones on each hind limb (Figure 1). 

The maximum temperature for each zone was used for analysis in line with previous infrared studies aimed at lameness detection in the cow [48,49]. 

### 2.4. Statistical Data Analyses

All data were analysed using SPSS version 25 (IBM Corporation, Armonk, NY, USA) except where stated. Descriptive statistics exploration was undertaken for each zone temperature measure. First, the normality of foot temperature was visually assessed using Q-Q plots and histograms. A generalised linear marginal repeated measures model was then used to evaluate the effect of the foot and zone within the foot on skin temperature. Foot (right or left hind) was the dependent variable, zone within foot the repeated variable and skin temperature the outcome variable. Covariance structure was identified using the Akaike information criterion. Residuals were checked for normality using Q-Q plots and histograms. Posthoc pairwise comparisons between marginal means were then used to compare between zones (with the Šidák correction for multiple comparisons [50]).

The association between locomotion score and foot temperature was tested using six temperature measures (summarised in Table 2). The relationship between these temperatures and locomotion scores was explored using box plots. This identified significant heteroscedasticity when foot temperature was compared across locomotion scores. Therefore, the association between foot skin temperature and locomotion score was analysed using a generalised linear model with an identity link function and robust estimators [51]. Each temperature definition was analysed as the outcome variable with LS as the predictor variable. 

A receiver operator characteristic (ROC) curve analysis was then performed. Six curves were created, one for each definition with categorised locomotion score (Lame (locomotion score ≥ 2) vs. not lame (locomotion score < 2)) to establish the sensitivity and specificity of IRT to predict locomotion score ≥ 2. The area under the curve (AUC) and coordinates of the curve (CC) were used to assess a model’s predictive accuracy. In addition, optimal threshold temperature cut-off values were determined by maximising sensitivity plus specificity. The statistical package software MedCalc Version 19.5.1 (MedCalc Software, Ostend, Belgium) was then used to calculate positive and negative predictive values for those optimal cut-offs.

## 3. Results

Data for both locomotion scoring and infrared thermography (430 thermograms, one per hind limb) were available from 215 cows from the 940-cow herd. 

### 3.1. Effect of Foot and Foot Zone on Skin Temperature

There was no evidence of a meaningful difference between feet in skin temperature; left and right foot mean temperatures were 33.37 °C (95% CI: 33.286–33.443) and 33.58 °C (95% CI: 33.512–33.657), respectively, with a mean difference of 0.21 °C (95% CI: 0.18–0.45). However, differences between zones were identified; the difference between the zone with the lowest mean temperature (zone 6) and the zone with the highest mean temperature (zone 4) was 1.11 °C (95% CI: 0.87–1.34).

**Figure 2:**
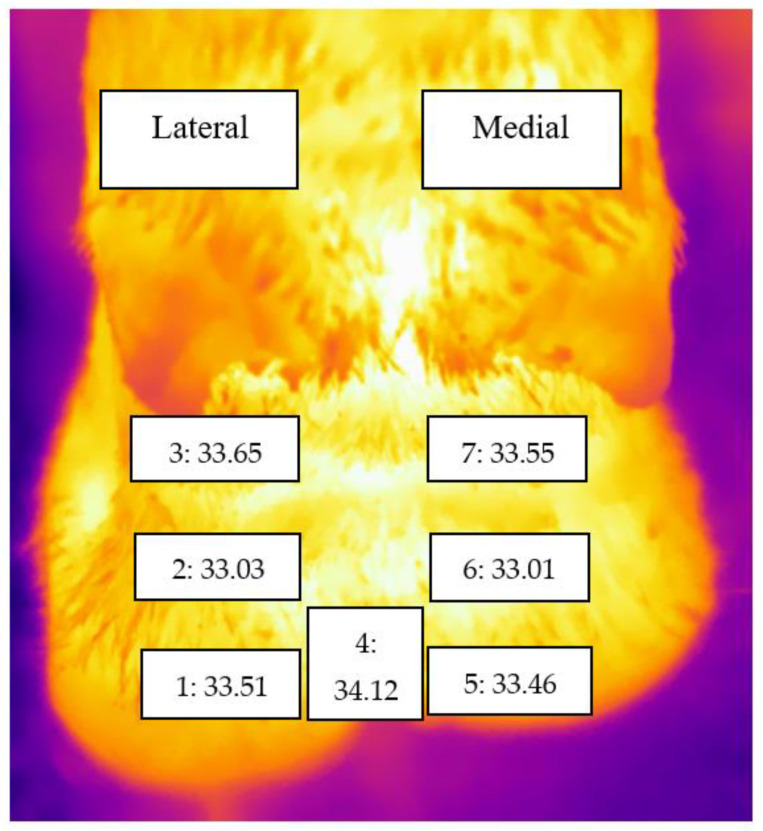
Thermogram (a plantar aspect of left hind foot) illustrating the mean temperature (degrees centigrade) for zones 1–7 in the left hindfoot. On the lateral claw; zone 1: coronary band, zone 2: above the coronary band, zone 3: below the accessory digit, zone 4: interdigital space, zones 5 to 7 are the equivalent to zones 1 to 3 but on the medial claw.

Although mean temperatures were higher for the zones on the lateral claw than the equivalent zones on the medial claw (see Table 3, Figure 2), these differences were small (between 0.02 and 0.1 °C).

#### 3.1.1. Infrared Thermography versus Locomotion Scoring 

Of the 215 cows with data from both infrared thermography and locomotion scoring, 86 had score 0 (40%), 99 had score 1 (46%), 27 had score 2 (12.6%), and 3 had score 3 (1.4%). Due to the low number of cows with a score of 3, the data from the cows with scores 2 and 3 were amalgamated as a score of ≥2. 

For all six temperature measures (Table 2), the temperature was higher for cows with a locomotion score of 1 than those with a score of 0 and higher for cows with a locomotion score of ≥2 than those with a score of 1. 

**Figure 3:**
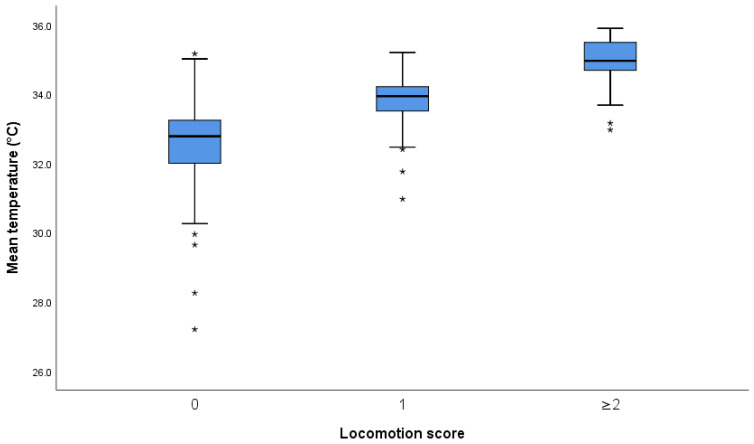
Boxplot of mean temperature (all zones, both feet) versus locomotion score (*n* = 215). Outliers are marked as asterisks.

**Figure 4:**
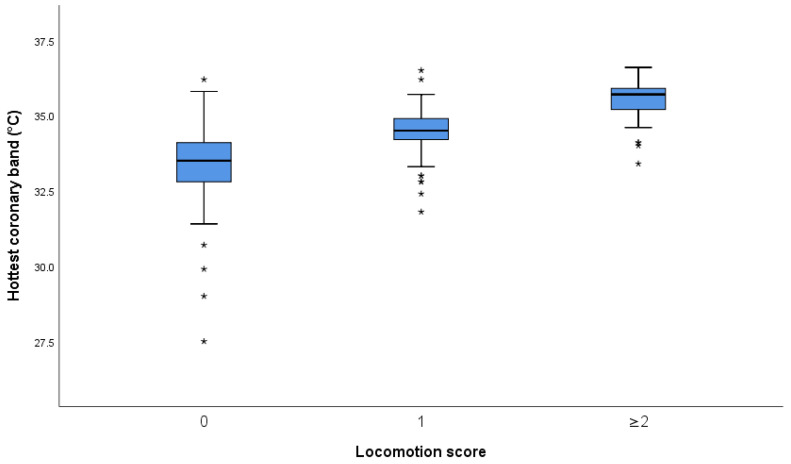
Boxplot of hottest coronary band temperature (zone 1 or 5) versus locomotion score (*n* = 215). Outliers are marked as asterisks.

Since the results for all zones showed the same trend, data are presented for mean temperature (MT) and the hottest coronary band zone (CB) only. The remaining data are presented in Appendix A. For MT, the mean difference between cows with scores 0 and 1 was 1.24 °C (95% CI: 0.9–1.58), and between scores 1 and ≥2 cows, it was 1.06 °C (95% CI: 0.58–1.54). The equivalent figures for CB were 1.2 °C (95% CI: 0.84–1.56) and 0.98 °C (95% CI: 0.47–1.49). The boxplots for MT and CB temperature measures are presented in Figure 3 and Figure 4, respectively.

**Figure A1:**
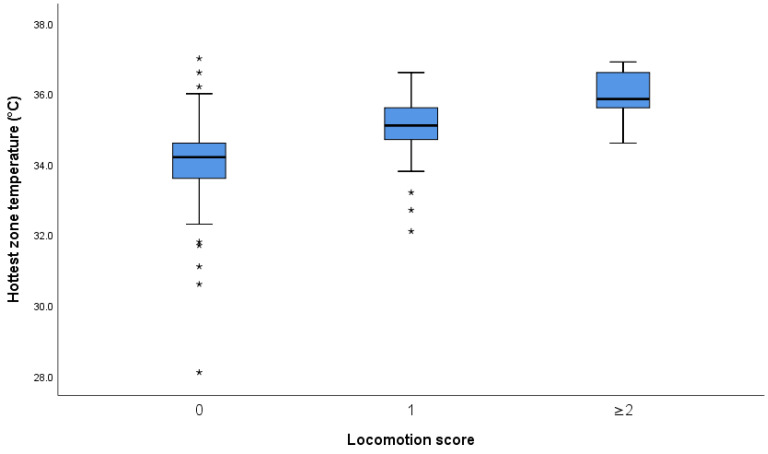
Boxplot of hottest zone temperature (out of all 14 zones) versus locomotion score (*n* = 215). Outliers are marked as asterisks.

**Figure A2:**
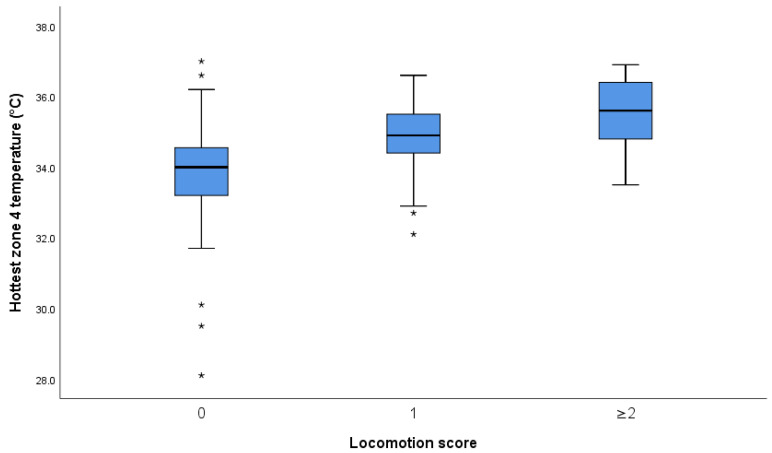
Boxplot of hottest zone 4 (interdigital space) versus locomotion score (*n* = 215). Outliers are marked as asterisks.

**Figure A3:**
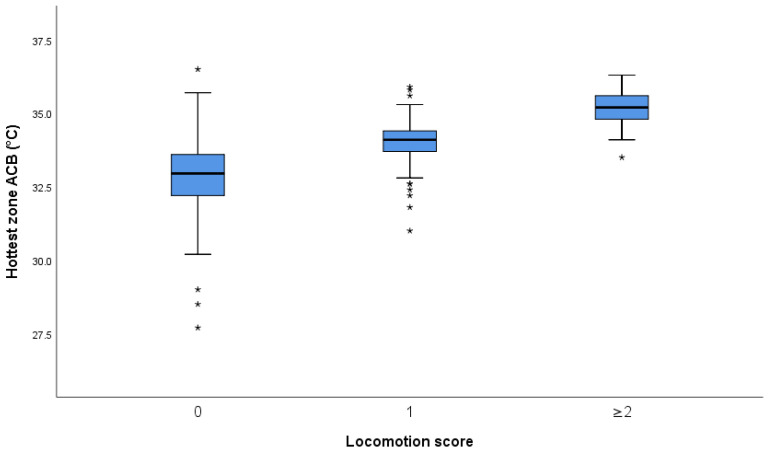
Boxplot of the hottest zone above the coronary band (zone 2 or 6) versus locomotion score (*n* = 215), ACB = above the coronary band. Outliers are marked as asterisks.

**Figure A4:**
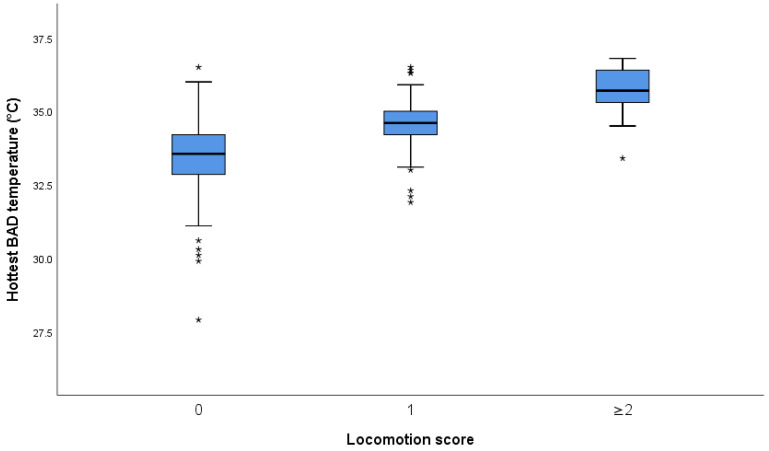
Boxplot of the hottest zone below the accessory digits (zone 3 or 7) versus locomotion score (*n* = 215), BAD = below the accessory digits. Outliers are marked as asterisks.

Interpretation Figure 3 and Figure 4 and Appendix B Figure A1, Figure A2, Figure A3 and Figure A4: The central box spans the quartiles, and the line in the box denotes the median. The line extends from the box (whiskers) to 1.5 times the interquartile range. Observations more than 1.5 times the interquartile range from the median are plotted individually as possible outliers (asterisks).

#### 3.1.2. Association of Foot Temperatures and Locomotion Scores

There was a linear association between individual cow locomotion score and foot skin temperature for all six temperature measures. The data for MT and CB are presented in Table 4 and Appendix A Table A1 for the other four temperature measures. For MT, each one-unit locomotion score increase (assuming LS ≥ 2 was LS = 2) was associated with a 0.944 °C (95% CI: 0.781–1.141) rise in mean temperature. For CB, every one-unit increase in locomotion score was associated with a 1.067 °C (95% CI: 0.883–1.289) increase in the hottest CB temperature. 

#### 3.1.3. A Receiver Operating Characteristic (ROC) Analysis

**Figure 5:**
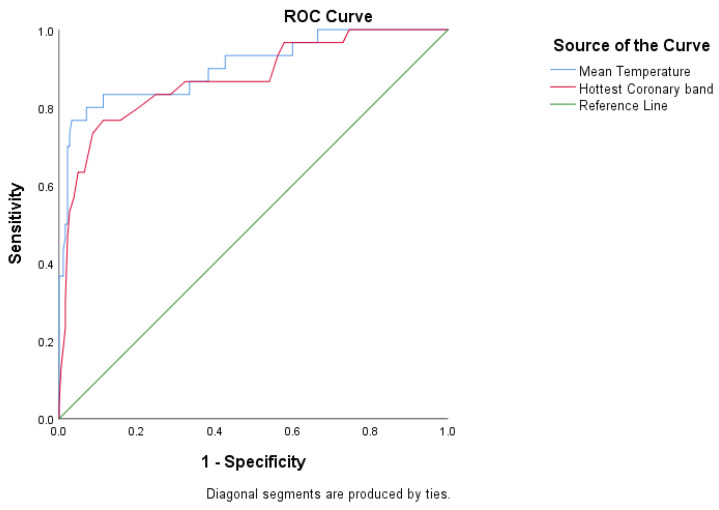
A receiver operating characteristic (ROC) curves; used to determine the optimal threshold values for the infrared thermography’s sensitivity and specificity, assuming locomotion scores ≥ 2 as locomotion score = 2 (*n* = 215).

**Figure A5:**
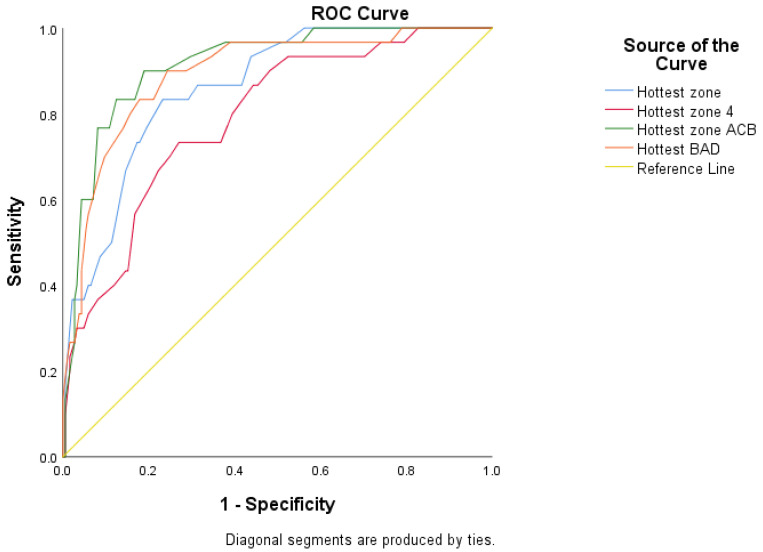
A receiver operating characteristic (ROC) curve; used to determine the optimal threshold values for the infrared thermography’s sensitivity and specificity, assuming locomotion scores ≥ 2 as locomotion score = 2 (*n* = 215). ACB = above the coronary band, BAD = below the accessory digits.

ROC curves for MT and CB are presented in Figure 5 (see Appendix B, Figure A5 for other temperature measures). In addition, optimal threshold values, area under the curve, and calculated parameters for MT and CB temperature measures are summarised in Table 5 (see Appendix A Table A2, for the results for other temperature measures).

## 4. Discussion

The present study aimed to evaluate the use of infrared thermography (IRT) as a tool for detecting lameness in a pasture-based dairy herd against the widely used visual locomotion scoring. IRT has been previously employed to detect foot lesions [32,36,40,44,48] and is associated with locomotion scores in housed cows [43,52]. However, the current study used IRT to detect gait changes (higher locomotion scores) in cows kept permanently at pasture. 

### 4.1. Feasibility of Infrared Thermography as a Method on New Zealand Dairy Farms 

For this study, thermal imaging of the plantar aspect of the foot was done alongside routine pregnancy diagnosis, without physical animal contact. However, even with the slowing down of the platform for pregnancy diagnosis, it was impossible to obtain an IRT image for every cow due to the time required to generate an image of the suitable quality of each foot. This is obviously a major limitation of the protocol, as to score an entire herd, IRT will need to be used on multiple occasions. However, in a pasture-based system, not all cows can be locomotion scored at one milking. The high flow rate of cows exiting the milking parlour makes it impossible to observe the gait of all cows and individually identify an observed cow’s number [27]. Nevertheless, cows that are not recorded as having a locomotion score are much more likely to have locomotion scores of 0 or 1 because it is much easier to detect and identify lame and severely lame cows exiting the milking parlour than cows with no or minor gait changes. Thus to be used as an alternative to locomotion scoring, IRT needs to be much faster than it is currently. An automated imaging process from a fixed point would be faster. However, there would be challenges regarding picture quality as there will be no camera repositioning if the foot is not in focus.

One limitation of this study is that the imaging process only captured temperature measurements of hind feet. Although, in housed cattle, hind limb lameness accounts for more than 90% of dairy cows [53], in New Zealand, the proportion of lame cows with hindfoot lesions is lower (71 and 56% in cows and heifers, respectively [54]). In New Zealand, the proportion of lame cows with front foot lesions is higher (29 and 44% in cows and heifers, respectively [54]). Thus only measuring hind feet is likely to have reduced the sensitivity of IRT for detecting lameness. However, front foot lameness may increase foot skin temperature in the hind feet as the animals compensate for that lameness by increasing the weight borne by the hind limbs. 

In addition, the IRT process may not detect cows that are lame due to non-hoof lesions (e.g., lesions of the hock or stifle). However, non-hoof-related lesions cause ~5% of lesions in lame dairy cattle in New Zealand, only [54]. Nevertheless, in the pasture-based production system that predominates in New Zealand, the only feasible time for collecting IRT images is during milking, when it is impossible to obtain high-quality images of the forelimbs easily and quickly. Therefore, further research on more cows and farms is required to establish how best to address these challenges and apply IRT in cows kept at pasture.

Cows’ feet were not washed before IRT, as Stokes et al. [48] found no clinically significant difference between cleaned and dirty feet when using IRT. Furthermore, cleaning the feet would have significantly increased the time taken to obtain IRT images, further decreasing the proportion of the herd which could be imaged per milking. Nevertheless, several researchers have cleaned the feet before IRT in their studies [48,55,56]. Hence further research on the value of washing before IRT in pasture-based cattle is needed, particularly whether the benefit of washing changes during the season as cow dirtiness changes.

### 4.2. Skin Foot Temperature and Effect of Claw and Zone

In the present study, we used maximum temperatures within each zone for all the analyses as recommended for detecting lesions with IRT [48,49,57]. In addition, both hindlimbs were evaluated together as lameness-causing foot lesions can occur across both hind feet with equal likelihood. We found that lateral claws had a higher mean temperature than medial claws, though differences were small (highest difference of 0.1 °C between zones 3 and 7) (Figure 2). Other studies have reported larger differences in temperature between lateral and medial claws [34,58]. For example, Nikkhah et al. [34] reported that the temperature difference between the coronary band and area above the coronary band was 5.2 °C and 4.2 °C for lateral and medial claws, respectively, while Wilhelm et al. [58] reported mean temperatures of 18.6 °C and 16.9 °C for lateral and medial claws, respectively. However, both these studies were on trimmed feet, and Nikkhah et al. [34] recorded the temperature of the dorsal wall while Wilhelm et al. [58] recorded the temperature of the solear surface. In contrast, a recent study by Gianesella et al. [56] reported higher medial claw temperatures than lateral claw temperatures in both healthy cows and those with claw lesions. They reported that the difference between medial and lateral claws was 2.3 °C and 2.1 °C for healthy and diseased claws, respectively.

Our results showed that both lameness and claw zone position (medial or lateral) affected temperature. These findings suggest that the small observed temperature difference between claws may be related to hindlimb lateral claws being more prone to claw horn lesions [34,54,59] and thus may have reflected subclinical conditions of the claws, which were not yet apparent. Further research is required to test this hypothesis. The effect of the zone was the same across claws, e.g., zones 1 and 5 (coronary band) had higher temperatures than zones 2 and 6 (skin above the coronary band). These results are consistent with previous studies [32,34,60,61]. However, the mean temperature difference in the current study is small, with 0.48 °C being the largest difference between the coronary band and skin above the coronary band. 

Temperature measurements for the other zones evaluated in the current study have not been frequently reported. However, considering the claws, zones 3 and 7 (below the accessory digits) had a higher temperature than both zones 2 and 6 (above the coronary band) and zones 1 and 5 (coronary band). Zone 4 (interdigital space) also had a higher temperature than the other zones within the foot. 

The higher skin temperature measured in the interdigital space (zone 4) could be explained by anatomical features. This hairless area is highly vascularised, and skin from both claws meets at this point; therefore, friction could be generated between the two claws in this relatively confined area leading to a rise in skin surface temperature. However, it is also a potential location for diseases such as foot rot, interdigital dermatitis, interdigital hyperplasia, and digital dermatitis [62]. Therefore, recording the temperature of zone 4 may be a more specific means of detecting those infectious diseases than measuring the temperature of other zones. However, this needs further investigation under New Zealand conditions because the farm was free of digital and interdigital dermatitis during this study and had a very low prevalence of footrot, so it was not suitable for testing this hypothesis.

### 4.3. Infrared Thermography as a Predictor of Locomotion Score

Lameness prevalence (scores ≥ 2) of the cows examined in the present study was 14%. Although the current study did not include the lame group, this finding is within the range of results reported by Fabian et al. [23], who reported that lameness prevalence on 59 dairy farms across New Zealand ranged from 1.2 to 36% (mean 8.1%). 

The present study revealed that median claw temperature increased as locomotion scores increased (Table 4). This is consistent with previous studies that have measured skin temperature in the same foot region as this study. For example, Lin et al. [52], who used a non-contact infrared thermometer on washed feet and measured the temperature of the skin in an area roughly equivalent to zones 2 and 6 in this study, reported that they were able to differentiate score 0 from score 1 and score 1 from ≥2 using their temperature measurements. However, not all results have been as clear. Rodríguez et al. [43], who used a thermal camera and measured skin temperature in the same area as Lin et al. [52], were able to separate cows with score 0 from cows with score 2 and score 3, but could not separate score 1 cows from cows with higher or lower locomotion scores. The mean skin temperatures recorded by [43] (after washing) were 20.2, 23.2, 24.8 and 25.9 °C for locomotion score 0, 1, 2, and 3, respectively, so this lack of differentiation may be a lack of power (as Rodríguez et al. [43] only had 30 cows per score group).

The present study used a cut-off of 34.5 °C for the mean temperature of all 14 zones, and this cut-off value maximised sensitivity and specificity at 80 and 92.4%, respectively (Table 5 and Appendix A Table A2). Thus, this cut-off point and the values for sensitivity and specificity are higher than previously reported figures by studies of IRT and LS. For example, Rodríguez et al. [43], using a cut-off of 25.5 °C, reported a sensitivity of 46.7% and specificity of 89.7%, while Lin et al. [52], using a cut-off of 23.3 °C, reported sensitivity of 78.5% and specificity of 39.2%. The sensitivity and specificity are also higher, though not so clearly than previous studies evaluating IRT and clinical lameness. For example, Main et al. [40], using a cut-off of 25.25 °C, reported a sensitivity of 72% and specificity of 73%, while Stokes et al. [48], with a cut-off of 27.0 °C, reported a sensitivity of 80% and specificity of 73%. 

Nevertheless, even the high specificity reported in this study is not sufficiently high for IRT to be used as the sole screening method for identifying cows that require lameness treatment. In this population, the positive predictive value of IRT was <65%, i.e., 1/3 of cows predicted as having a locomotion score > 1 by IRT actually had a score ≤ 1. If cows identified as lame by IRT are progressed to lameness treatment without further observation such as LS, a significant amount of staff time will be wasted examining non-lame cows. If IRT were to be used for frequent, ongoing monitoring in a population (e.g., daily measurement), this issue would be even more significant as monitoring will reduce the number of unidentified lame cows. Therefore, increase the proportion of the herd that is not lame, and the proportion of false positives produced by IRT. For example, in a herd where no cows are lame, IRT will still identify on average 15 lame cows for every 200 cows examined (specificity = 92.4%). 

One caveat to this discussion is that the calculation of sensitivity and specificity used in this study assumes that locomotion scoring is a gold standard when it is known that LS does not have 100% specificity or sensitivity [21,63,64]. This could be addressed by using a latent class analysis which does not assume that either LS or IRT are a gold standard. However, the authors are not aware of any latent class analysis of LS, and such an analysis is beyond the scope of this paper.

The comparison of specificity and sensitivity results highlighted the difference between the optimal temperature thresholds identified in this study and previous studies. Previous studies of IRT and LS and IRT and clinical lameness have identified a range of thresholds [36,40,43,44,48,52,55,56], but, as far as the authors are aware, this study’s optimal cut-off is the highest reported. The difference between the threshold in this study and the highest previously reported threshold is much greater than the differences between previous studies. Some of this can be explained by differences in the protocol; e.g., both Rodríguez et al. [43] and Lin et al. [52] measured skin temperature after washing, but washing reduces temperatures by up to 2 °C [48], not than the 8–10 °C difference seen between the thresholds identified in the current study and those of previous studies. Thus other factors must be influencing at least some of the differences. These may include ambient temperatures, e.g., studies in the UK [36,40,48,62] were undertaken during the autumn/winter/spring seasons, while our study was conducted during summer. However, it is also possible that a key reason for the difference is that the cows in this study were all kept at pasture, whereas previous studies were undertaken in housed cows. Perhaps the main difference between these two systems is that cows at pasture are much more active in contrast to housed cows. In particular, the cows in this study will all have recently walked from the grazing area to the milking parlour. There have been no published data on the impact of such activity on foot skin temperature, but it is likely to have increased blood flow and, therefore, skin temperature. Further research is needed to understand better how walking affects temperature as there is significant variation in distance walked from pasture to the milking parlour within and between farms.

Nevertheless, even though we do not know exactly how walking distance affects foot temperature. It is another variable that needs to be considered when interpreting IRT results alongside other factors such as ambient temperature, recent rainfall, current weather, foot cleanliness, and lactation stage. All of these factors change day-to-day, and thus their effect could not be investigated in this study which was based on the analysis of results from a single timepoint. There are also likely to be significant differences between farms in many of these factors. It is thus likely that the optimal threshold temperature for IRT in cattle kept at pasture will not be consistent across farms or over time within a single farm. Further research across New Zealand on more farms for longer periods is required to identify how optimal IRT threshold changes and the key factors responsible for this change.

If thermal scanning does become feasible as an on-farm lameness detection method, its use will probably have to be based on repeated measurements on individual cows over time (which will necessitate some form of automation). Furthermore, these repeated IRT results will have to be combined with multiple inputs from other sources (such as weather stations). This practice will create a large dataset that is best analysed using a machine learning type process that can deal with within and between farm heterogeneity (such as classification by analysis which has just been used for diagnosing mastitis from a similarly complex dataset [65]). 

## 5. Conclusions

Our results demonstrated that the plantar aspect of the hindfoot could be easily thermally imaged for measuring the hindfoot skin temperature. Therefore, this location can be used for assessing the presence of foot-associated lameness-causing lesions as it can evaluate multiple anatomical areas, including coronary band, surface skin above CB, interdigital space, and surface skin below the accessory digit. Furthermore, the results of the present study show that such measurements can be used to distinguish between cows with different locomotion scores such as score 0 (sound–cows that do not need attention with regards to lameness), 1 (imperfect gait–cows that need close observation), and ≥2 (lame cows that need treatment). Therefore, IRT has a considerable potential to be used on-farm to screen for lameness. However, the specificity of IRT observed in the current study does not appear high enough for IRT to be used as an alternative to locomotion scoring [66]. In addition, automation of the process will likely be necessary for IRT to be used without interfering with farm operations. This automation will also open the way for repeated skin temperature measurements, resulting in more accurate lameness detection than single measurements, especially if the IRT data are combined with other inputs in a machine learning process.

## Figures and Tables

**Table 1:** The gait and posture attribute used during locomotion scoring with the DairyNZ system [45].

Score	Clinical Term	Evaluation Criteria
0	Sound	The cow walks confidently, even weight-bearing and tracks up.
1	Imperfect locomotion	The cow walks unevenly, does not track up, with a mildly arched back when walking.
2	Lame	An arched back, the favoured limb moves faster than the lame leg, feet placed unevenly, head bobs up and down when walking.
3	Severely lame	Walks very slow, reluctant to bear weight, arched back, and head bobs obvious.

**Table 2:** Definitions of the infrared thermography estimates utilised for analysis.

Foot/Zone *	Description
Mean temperature	Average temperature, across both feet (all 14 zones)
Hottest zone	Highest zone temperature, across both feet (all 14 zones)
Hottest zone 4	The highest zone 4 temperature on either foot
Hottest coronary band (CB)	The highest zone 1 or 5 temperature on either foot
Hottest above the coronary band zone (ACB)	The highest zone 2 or 6 temperature on either foot
Hottest zone below the accessory digit (BAD)	The highest zone 3 or 7 temperature on either foot

* For all analyses, the maximum temperature for each zone was used for the analysis.

**Table 3:** The mean temperature and 95% confidence interval for zones 1–7 (see Figure 2) for both hindlimbs (430 feet) of 215 cows (degrees centigrade).

Zone	Mean	95% Confidence Interval	
		Lower Bound	Upper Bound
1	33.51	33.375	33.646
2	33.03	32.889	33.165
3	33.65	33.507	33.787
4	34.12	33.986	34.250
5	33.46	33.323	33.596
6	33.01	32.869	33.148
7	33.55	33.414	33.691

**Table 4:** Temperature measures estimates and their 95% confidence intervals (*n* = 215).

Model Parameter		95% Confidence Interval	
		Lower Bound	Upper Bound
Mean temperature (intercept)	34.877	34.614	35.140
Locomotion score *	0.944	0.781	1.141
Hottest coronary band (intercept)	35.483	35.199	35.768
Locomotion score *	1.067	0.883	1.289

* Effect of increase in locomotion score of 1 unit in DairyNZ lameness score (all score 3 cows recorded as score 2). See Table 2 for the definition of temperature measurement.

**Table 5:** Optimal cut-off points for skin foot temperature measurements (degrees centigrade) for determining lame cows (Dairy NZ lameness score ≥ 2).

Temperature Measure ^1^	Optimal Threshold (°C)	AUC (95% CI)	Specificity (95% CI)	Sensitivity (95% CI)	PPV (95% CI) *	NPV (95% CI) *
Mean Temperature	34.5	0.91 (0.84–0.97)	92.4 (87.63–95.80)	80.0 (61.43–92.29)	63.3 (50.21–74.60)	96.6 (93.27–98.31)
Hottest coronary band	35.1	0.87 (0.80–0.95)	85.4 (79.48–90.16)	76.7 (57.72–90.07)	46.1 (36.42–56.07)	95.7 (92.14–97.73)

^1^ See Table 2 for the definition of temperature measure; AUC, area under the curve; CI: confidence interval; PPV, positive predictive value and NPV, negative predictive value; (*, PPV and NPV calculated at a prevalence of 14%) analysis based on data from 215 cows.

## Data Availability

Data are available at request from the corresponding author.

## Appendix A

**Table A1:** Temperature measures estimates and their 95% confidence intervals (*n* = 215). Illustration: Hottest zone above the coronary band (ACB), hottest zone below the accessory digit (BAD) temperatures (*n* = 215).

Model Parameter		95% Confidence Interval	
		Lower Bound	Upper Bound
Hottest zone (intercept)	35.957	35.716	36.197
Locomotion score *	0.958	0.793	1.157
Hottest zone 4 (intercept)	35.590	35.280	35.900
Locomotion score *	1.218	1.008	1.471
Hottest ACB (intercept)	35.153	34.915	35.392
Locomotion score *	1.087	0.900	1.314
Hottest BAD (intercept)	35.657	35.385	35.929
Locomotion score *	1.126	0.932	1.361

* Effect of increase in locomotion score of 1 unit in DairyNZ lameness score (all score 3 cows recorded as score 2). See Table 2 for the definition of temperature measurement.

**Table A2:** Optimal cut-off points for skin foot temperature measurements (degrees centigrade) for determining lame cows (Dairy NZ lameness score ≥ 2). Illustrations: ^1^ See Table 2 for the definition of temperature measure; AUC, area under the curve; CI: confidence interval; PPV, positive predictive value and NPV, negative predictive value; (*, PPV and NPV calculated at a prevalence of 14%) analysis based on data from 215 cows.

Temperature Measure ^1^	Optimal Threshold (°C)	AUC (95% CI)	Specificity (95% CI)	Sensitivity (95% CI)	PPV (95% CI) *	NPV (95% CI) *
Hottest zone	35.7	0.861 (0.799–0.923)	82.2 (75.87–87.39)	73.3 (54.11–87.72)	40.1 (31.46–49.39)	95.0 (91.24–97.17)
Hottest zone 4	35.2	0.790 (0.707–0.873)	72.4 (65.40–78.73)	73.3 (54.11–87.72)	30.2 (23.96–37.31)	94.4 (90.15–96.82)
Hottest zone ACB	35.1	0.914 (0.865–0.963)	95.7 (91.66–98.11)	60.0 (40.60–77.34)	69.3 (51.92–82.53)	93.6 (90.45–95.80)
Hottest zone BAD	35.1	0.893 (0.832–0.954)	82.2 (75.87–87.39)	83.3 (65.28–94.36)	43.2 (34.93–51.86)	96.8 (93.13–98.54)
